# Gamma and Beta Oscillations in Human MEG Encode the Contents of Vibrotactile Working Memory

**DOI:** 10.3389/fnhum.2017.00576

**Published:** 2017-12-04

**Authors:** Alexander H. von Lautz, Jan Herding, Simon Ludwig, Till Nierhaus, Burkhard Maess, Arno Villringer, Felix Blankenburg

**Affiliations:** ^1^Neurocomputation and Neuroimaging Unit, Department of Education and Psychology, Freie Universität Berlin, Berlin, Germany; ^2^Bernstein Center for Computational Neuroscience Berlin, Berlin, Germany; ^3^Max Planck Institute for Human Cognitive and Brain Sciences, Leipzig, Germany

**Keywords:** working memory, MEG, somatosensory, gamma, beta, oscillations

## Abstract

Ample evidence suggests that oscillations in the beta band represent quantitative information about somatosensory features during stimulus retention. Visual and auditory working memory (WM) research, on the other hand, has indicated a predominant role of gamma oscillations for active WM processing. Here we reconciled these findings by recording whole-head magnetoencephalography during a vibrotactile frequency comparison task. A Braille stimulator presented healthy subjects with a vibration to the left fingertip that was retained in WM for comparison with a second stimulus presented after a short delay. During this retention interval spectral power in the beta band from the right intraparietal sulcus and inferior frontal gyrus (IFG) monotonically increased with the to-be-remembered vibrotactile frequency. In contrast, induced gamma power showed the inverse of this pattern and decreased with higher stimulus frequency in the right IFG. Together, these results expand the previously established role of beta oscillations for somatosensory WM to the gamma band and give further evidence that quantitative information may be processed in a fronto-parietal network.

## Introduction

The ability to maintain behaviorally important sensory information over short periods of time is a key component of working memory (WM). The neural basis of this cognitive function has been attributed to the lateral prefrontal cortex (PFC), whose neural firing rates are modulated during stimulus retention (for review, see D’Esposito, 2007). Research in the somatosensory domain provides evidence that single neurons in the PFC can encode WM content by monotonically increasing and decreasing their firing rate (Romo et al., 1999; Brody et al., 2003). In these studies responses of neurons from the right inferior convexity of the PFC were recorded in behaving monkeys trained to decide whether the second (f2) of two sequentially presented frequencies was higher or lower than the first (f1). Hence, this task requires remembering f1 throughout a short retention interval between both stimuli. Firing rates observed during this retention interval changed as a function of f1 and were directly related to behavior, in line with an interpretation as a neural substrate of parametric WM (for review, see Romo and de Lafuente, 2013).

Complementing these findings from non-human primates, human electroencephalography (EEG) recordings during the same task have revealed a parametric increase of oscillatory power in the beta band (15–35 Hz) as a function of f1 (Spitzer et al., 2010; Spitzer and Blankenburg, 2012). The source of this modulation was consistently found in the right inferior frontal gyrus (IFG) of the PFC. Expanding on these findings, Spitzer and Blankenburg (2012) and Spitzer et al. (2014) demonstrated this effect across sensory modalities and stimulus features, indicating a generalized role of prefrontal beta oscillations for maintaining quantitative information.

Magnetoencephalography (MEG) studies on the other hand have identified modulations of high frequency gamma oscillations (>40 Hz) accompanying somatosensory WM (Bauer et al., 2006; Haegens et al., 2010). In a vibrotactile delayed match-to-sample task, Haegens et al. (2010) demonstrated that relative to a pre-stimulus baseline, gamma power increased during the WM interval in the secondary somatosensory (SII) and frontal cortices. Furthermore, the frontal power increase correlated positively with behavioral performance, suggesting a functional role for gamma oscillations around 65–80 Hz. These results corroborate findings from other sensory domains (for reviews, see Benchenane et al., 2011; Roux and Uhlhaas, 2014; Lara and Wallis, 2015) and intracranial recordings in monkeys (Pesaran et al., 2002). Specifically, MEG studies in humans have shown that visual and auditory WM is accompanied by sustained gamma band activity in modality specific sensory areas (Lutzenberger et al., 2002; Kaiser et al., 2003; Jokisch and Jensen, 2007).

However, the available evidence for an involvement of high frequency oscillations in somatosensory WM is limited to contrasting periods of high vs. low WM load. Indeed, while investigations into the functional role of the beta-band demonstrated a parametric mapping of stimulus identity to oscillatory power, the role of gamma in maintaining stimulus features remains unclear.

In the present study, we investigated the role of cortical oscillations for the parametric encoding of human somatosensory WM. Subjects performed a vibrotactile frequency comparison task with stimuli consisting of different frequencies delivered to the left index finger. The neural substrates of performing this task were measured non-invasively with whole-head MEG, allowing for the tracking of fast oscillatory changes in high frequencies. We hypothesized that in addition to the well-established modulation of frontal beta band power by f1, oscillations in the gamma band would also be modulated by the to-be-maintained stimulus frequency.

## Materials and Methods

### Participants

Twenty-three healthy volunteers (12 females, 23–37 years of age, median: 28) participated in the study and underwent a 30-min behavioral training session to learn the task one week before the MEG recording. All participants reported being right-handed, according to the Edinburgh Handedness Inventory (Oldfield, 1971), having no history of neurological illness and normal or corrected-to-normal vision. Volunteers provided written informed consent as approved by the local ethics committee of the Freie Universität Berlin in accordance with the Human Subjects Guidelines of the Declaration of Helsinki.

### Experimental Paradigm

**FIGURE 1:**
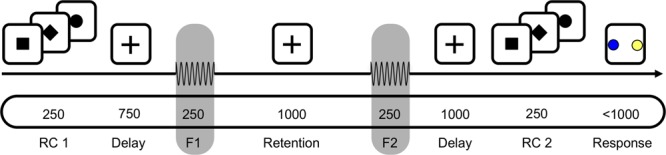
A schematic of the task for one example trial. First, a response cue (RC1) was presented for 250 ms to indicate whether to answer by saccade or button press. In half the trials a circle appeared instead, which indicated that the response modality would be indicated just before responding. After a 750 ms delay, the base frequency f1 was presented for 250 ms, followed by a 1000 ms retention interval. After presentation of the comparison frequency f2, the decision was delayed for 1000 ms until a second response cue (RC2) was shown for 250 ms. Participants responded by selecting one of the two colored targets, e.g., blue for f2 > f1, by saccade or button press. In the schematic, the top section depicts visual input; the middle the tactile; and the bottom the timing (in ms) of input.

Participants were asked to decide whether the second of two sequentially presented vibrotactile frequencies was higher or lower than the first, either by making a saccade to a visual target or by selecting the target via button press (**Figure 1**). Each trial started with a fixation cross being presented at the center of a screen in front of the participant for a variable duration (750–1250 ms) at a viewing distance of 90 cm. The response type (saccade or button press) for a given trial was indicated subsequently by a square or diamond presented at the location of the fixation cross for 250 ms (first response cue, RC1; **Figure 1**). Alternatively, in 50% of trials, a circle appeared at this time, indicating that the response mapping would only be disclosed via a second response cue just before participants were allowed to respond (RC2; **Figure 1**). Then, the two vibrotactile flutter stimuli (with frequencies 11–31 Hz) were briefly presented to the left index finger (250 ms each), separated by 1000 ms. The frequency of the first stimulus (f1) was varied between 15 and 27 Hz in steps of 4 Hz while the frequency of the second stimulus (f2) was either 2 or 4 Hz higher or lower than f1. The f2 presentation was followed by a delay of 1000 ms, after which the second response cue was presented for 250 ms (RC2; **Figure 1**). If the first response cue had already provided the response mapping (i.e., RC1 = diamond or square), a circle was presented. In case the first response cue was uninformative (i.e., RC1 = circle), the second response cue revealed whether participants should respond via button press or saccade (i.e., RC2 = diamond or square). Following this, two colored target dots were presented at the left and right side of the screen with eccentricity of 12° visual angle (‘go’-cue). One dot was blue, and the other one yellow, with the specific spatial configuration being counterbalanced across trials (i.e., blue dot was equally likely on either side). Each participant applied one of two possible color mappings (i.e., if f2 > f1, choose blue; if f2 < f1, choose yellow, or vice versa) that were counterbalanced across participants, and selected one of the colored dots according to their decision as soon as the target dots appeared (i.e., either by button press or saccade, depending on the cued response modality).

Partipants completed six blocks with 128 trials each. Within each block, half the trials were answered by button press, the other half by saccades (64/64). Similarly counterbalanced was the position of the informative response cue, with half the response types indicated before and the other half after stimulus presentation (64/64). The total of 768 trials per participant resulted in a scanning time of about 75 min.

### Stimuli

All stimuli were created using a PC running the MATLAB-based Psychophysics toolbox (Brainard, 1997). Vibrotactile stimulation was delivered by a piezoelectric Braille stimulation device (QuaeroSys, Schotten, Germany) to the left index finger. The 16 pins of the 4 × 4 Braille display were driven by a constant 121 Hz carrier signal whose amplitude was modulated by sinusoids with frequencies between 11 and 31 Hz, resulting in a percept of vibrotactile flutter at the modulation frequency (Tobimatsu et al., 1999). The stimuli were loaded into the buffer of the Braille stimulation device 1 s before the presentation of f1, as the communication of PC and Braille stimulator created noticeable artifacts. To mask the noise of the Braille display, white noise was replayed at 66 dB from electromechanical transducers and transmitted via sound conducting tubes to the ears inside the MEG helmet.

### Data Acquisition

Participants were positioned upright in the MEG system with their arms placed comfortably on a table in front of them. They were instructed to keep fixation on the presentation screen and not to move during the experiment. Magnetoencephalography was recorded using a whole-head MEG Vectorview NM2169N (Elekta Neuromag Oy, Helsinki, Finland) with a total of 306 MEG channels (102 magnetometers, 204 planar gradiometers). A band-pass filter of 0.03–500 Hz was applied during acquisition at 1500 samples/second and five head position indicator (HPI) coils attached to the scalp, three on the forehead and one on each mastoid, tracked the head movements continuously. Three fiducials (nasion, left and right preauricular points) as well as over 500 scalp points were measured with a Polhemus FASTRAK 3D digitizer to obtain the head shape of each participant. We did not employ electrooculography, because initial tests revealed that electrodes placed on the head increased artifacts from the QuaeroSys stimulation device (cf. Chandler et al., 2015).

Participants’ responses were tracked via a NNL-Response Grip from Nordic Neuro Lab (BNC – serial port) and an iView X MEG eye-tracking system (SensoMotoric Instruments GmbH, Berlin, Germany) sampled at 50 Hz. Saccades to the left or to the right further than nine degrees off-center were interpreted as a response to the according side. Trials in which participants showed lateral eye movements before the colored targets appeared and those in which the wrong response modality was used were excluded from further analysis. Before each block started, the eye-tracker was calibrated and validated with a standard five-point procedure.

### Data Processing

All MEG data were preprocessed using the Oxford Centre for Human Brain Activity software library (OSL)^1^ drawing on the Fieldtrip toolbox^2^ (Oostenveld et al., 2011) and SPM12 (Wellcome Department of Cognitive Neurology, London, United Kingdom ^3^).

As a first preprocessing step, we identified noisy channels and periods of strong artifacts by visually inspecting the continuous recordings. Then, using the MaxMove software (Elekta Neuromag), noise sources outside the skull were removed by applying signal-space separation with its temporal extension. Head movement compensation based on continuous tracking of the HPI coils was used and each individual’s data transformed to the co-ordinate frame of their third scanning block. Subsequently the continuous data were bandpass filtered at 0.1–165 Hz, down-sampled to 512 samples per second and cut into epochs with respect to f1 onset in a time window of -1000 to +1500 ms. After visual inspection of individual trials to identify extreme muscle artifacts, squid jumps and signal drop out, an independent component analysis (ICA), as implemented in the EEGLAB toolbox (Delorme and Makeig, 2004), was calculated to identify blink, saccade and heart beat components, which were excluded in the remixing of the data. We conservatively rejected only those components that showed a very typical artifactual nature. In a final visual inspection, trials with persisting artifacts were manually removed.

To obtain a time-frequency (TF) representation of spectral power we used a sliding window Fourier transform at steps of 20 ms and applied a Hann taper with seven cycles length for frequencies 5–40 Hz. For higher frequencies, we used a multitaper Fourier transform with a fixed sliding window of 200 ms and ±10 Hz smoothing.

Evoked power was calculated for each f1-f2 stimulus pair by computing the TF representation of the according event related fields (ERFs). ERFs were obtained by averaging all baseline-corrected trials (with respect to 650–150 ms prior to f1) for each stimulus pair in the time domain. Induced power was calculated by subtracting the ERFs of each stimulus pair from according single trials before transforming the single-trial data into the TF domain. The resulting single-trial TF representations were averaged for each condition (i.e., per stimulus pairs) to yield estimates of average induced power per condition. Finally, we applied a frequency-specific baseline correction by subtracting the average power in each frequency band 650–150 ms before f1 onset from the whole trial. For further analyses and display purposes, we combined the set of two orthogonal gradiometers at each location, resulting in 102 rectified planar gradiometers.

### Statistical Analysis

Time-frequency maps were convolved with a 3 Hz × 300 ms Gaussian smoothing kernel (Kilner et al., 2005) to reduce variability between trials. To investigate parametric coding of f1 frequency during the retention interval, we implemented a general linear model (GLM) with a one-factorial repeated measures design for individual trials with the four f1 conditions as factor levels (i.e., f1 = 15, 19, 23, or 27 Hz). The accordingly estimated parameter maps (beta images) were weighted with a zero-mean contrast vector of [-0.75, -0.25, 0.25, 0.75]. The resulting contrast images depict the parametric difference across the four conditions in each TF bin.

These images from all individuals were statistically validated via a cluster-based permutation test procedure over all subjects (Maris and Oostenveld, 2007). This test controls the false-alarm rate by using a cluster statistic that is evaluated under a permutation distribution of summary statistics of the observed data, which we established with 5000 randomly sign-flipped permutations. A cluster was defined as a group of adjacent time-frequency bins whose cluster-defining threshold surmounted *p*_threshold_ < 0.05. Clusters exceeding the family-wise error (FWE) corrected threshold of *p_FWE_* < 0.05 (corrected for time, frequency, and channels) were considered to be statistically significant. Cluster-based inference, which serves to reject the null hypothesis of the whole time-frequency-channel window, was supplemented by conventional linear trend analysis over time, pooled over the channels and frequency bands in which a significant effect had been observed. The aforementioned analysis steps were also applied to equal-sized subsets of correct and incorrect trials. For each cluster, the statistical comparisons were then based upon those channels and frequencies exhibiting a significant effect in the main parametric contrast of induced power. This cluster analysis was supplemented by conventional *t*-tests between correct and incorrect trials on all timepoints where significant clusters had been identified and were subjected to Bonferroni–Holm correction.

To maximize the power of these parametric contrasts, we pooled trials over both response modalities (i.e., saccades and button presses) and response cues (i.e., before and after stimulus presentation). To ensure that there were no differences between the underlying subgroups for the parametric WM effects, we applied the same procedure for these separate conditions. Moreover, to verify that response times (RTs) – as a measure of WM load – did not have an influence on the parametric coding of vibrotactile frequencies, we contrasted the four estimated parameter maps from the GLM inversion (i.e., one beta image for each base frequency) by the according individual mean RTs, instead of the actual f1 frequencies as in the main analysis. Both control analyses did not reveal any significant clusters during the WM period of this task.

### Source Reconstruction

The 3-D sources of the observed effects at the sensor level were reconstructed using T1-weighted structural magnetic resonance (MR) images. The images were acquired with a Siemens 3.0 Tesla TIM Trio or Verio scanner, either using a T1-weighted MPRAGE sequence (TR = 2300 ms, TE = 2.96 ms, flip angle = 9°, FOV = 256 mm × 240 mm × 176 mm, voxel size = 1.0 mm isotropic) or a T1-weighted MP2RAGE sequence (TR = 5000 ms, TE = 2.92 ms, TI1 = 700 ms, TI2 = 2500 ms, flip angle 1 = 4°, flip angle 2 = 5°, matrix size = 240 × 256 × 176, voxel size = 1.0 mm isotropic). The individual structural MR images were used to create cortical meshes of 8196 vertices by warping meshes from a brain template to the inverse spatial normalization of individual brains. The MEG recording sites were co-registered with the MRI using three fiducials: the nasion as well as the left and right pre-auricular points. The forward model (i.e., leadfield matrix) was estimated as a realistic single shell (Nolte, 2003).

The inversion of the forward model was based on the preprocessed MEG data in the time domain, prior to TF transformation. Before model inversion, the time domain signal was bandpass-filtered and epoched to representative time-frequency windows that reflected the features of the sensor space analysis; namely the significant times and frequencies of the cluster-based permutation test for the localization of the WM effect, and the time of f1 presentation in combination with according frequency bands (i.e., frequency of f1 ±1 Hz) for the localization of somatosensory steady-state evoked fields (SSEFs). The forward model was inverted using multiple sparse priors (MSP; Friston et al., 2008) under group constraints (Litvak and Friston, 2008) as implemented in SPM12 for each condition separately. For each participant, the results of model inversion were summarized by 3-D images reflecting the spectral source amplitude averaged over the corresponding TF windows of interest. These matched the significant clusters of the sensor level analysis for the WM effect, and were according to time and frequency of f1 presentation for the localization of SSEFs. For the source reconstruction of the WM effect, the summary images were contrasted in analogy to the sensor space analysis, namely by a parametric contrast corresponding to the four different f1 values (i.e., f1 = 15, 19, 23, 27 with contrast vector = [-0.75, -0.25, 0.25, 0.75]). For the source reconstruction of SSEFs, the 3-D summary images of spectral source power during f1 presentation (at corresponding frequencies) were weighted by the individual amplitudes of SSEFs as observed at the sensor level. Since somatosensory SSEFs (i.e., somatosensory steady-state evoked potentials recorded with EEG) are known to show a bell-shaped amplitude profile over stimulus frequencies in the flutter range when recorded at the scalp (e.g., Snyder, 1992; Tobimatsu et al., 1999), this specific amplitude profile was also used to identify the most likely cortical sources of SSEFs. On the group level, individual source estimates were contrasted using conventional *t*-tests. Sources that exceeded a statistical threshold of *p* < 0.01 (*p* < 0.001 for SSEFs; both uncorrected) were displayed to indicate the most likely sources underlying the effects observed at the sensor level. References to anatomical landmarks were established with the SPM anatomy toolbox (Eickhoff et al., 2005) and are expressed in the Montreal Neurological Institute and Hospital (MNI) coordinate system.

## Results

### Behavior

Participants correctly discriminated on average 69% (*SD* = 7%, **Table 1**) of all presented stimulus pairs and each participant’s correct responses exceeded the guess rate of 50%. A within-subjects ANOVA with the factors ‘base stimulus frequencies’ in Hz (15, 19, 23, 27) and ‘difficulty’ (±4 Hz vs. ±2 Hz) was performed on percentages of correct responses (PCR), logit-transformed to account for the non-normality of the residuals. This analysis revealed no effect of base stimulus frequency (i.e., f1) on the percentage of correct responses [*F*(3,66) = 1.25, *p* > 0.05]. However, as expected, participants were more successful on easy trials (f2-f1 = ±4 Hz) as compared to difficult trials [f2-f1 = ±2 Hz; *F*(1,22) = 101.64, *p* < 0.001]. Similarly, we performed a 2 × 2 within-subjects ANOVA with factors ‘response type’ (button vs. saccade) and ‘response cue’ (before vs. after stimulus presentation) on the logit transformed PCRs, which revealed no significant differences (all *p* > 0.05, see **Table 1**).

**Table 1:** Average task performance.

Behavioral performance		
**Frequency (Hz)**	**% Correct**	**RT (ms)**
15	67 (12)	437 (106)
19	69 (9)	430 (97)
23	71(9)	428 (93)
27	66 (7)	425 (93)
**Total**	**69 (7)**	**430 (97)**
*f1-f2 (Hz)*		
–4	69 (11)	432 (98)
–2	62 (9)	433 (101)
2	65 (9)	435 (100)
4	79 (11)	420 (90)
**Response cue before**		
Button press	69 (8)	379 (109)
Saccade	69 (7)	443 (91)
**Response cue after**		
Button press	68 (8)	419 (110)
Saccade	69 (8)	478 (99)

*The *top* part shows the performance for the four base (f1) frequencies as proportion of correct responses (PCRs; in %) as well as the median reaction times (RTs) in milliseconds. The *middle* part depicts PCRs and RTs as a function of the difference between base (f1) and comparison (f2) frequency. The *bottom* part shows the performance for the different response modalities, separate for whether the response cue appeared before or after vibrotactile stimulation. All entries are followed by the corresponding standard deviation in brackets.*

On average, participants responded 430 ms after the ‘go’-cue, i.e., after displaying the response mapping on the screen. Because we applied a forced-delay decision task, RTs were not expected to show large variability across different stimulus conditions. Accordingly, a within-subjects ANOVA with factors ‘base stimulus frequencies’ and ‘difficulty’ of the median RTs did not reveal any significant differences (all *p* > 0.05, see **Table 1**). The same analysis with the factors ‘response type’ and ‘response cue’ showed faster answers by button press than saccades [*F*(1,22) = 24.82, *p* < 0.001]. One reason for this difference was that detecting saccades accurately was slower than reading out button presses. Participants also gave faster responses when the response cue was delivered before stimulus presentation [*F*(1,22) = 30.71, *p* < 0.001, for a list of all RTs see **Table 1**].

### Stimulus-Evoked Fields

**FIGURE 2:**
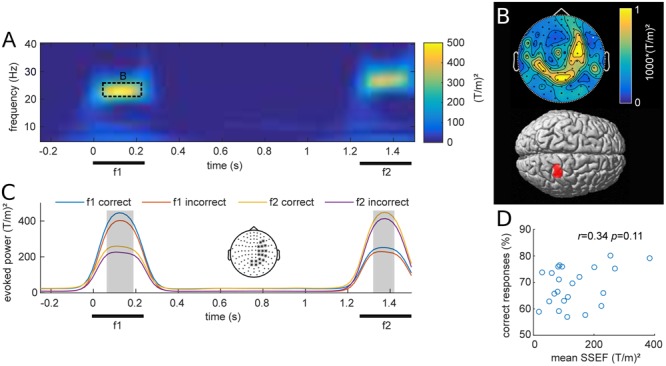
**(A)** Grand average of stimulus evoked fields over all participants and planar gradiometers for an example stimulus pair (base = 23 Hz; comparison = 27 Hz). The steady-state evoked field (SSEF) at the time of f1 and f2 stimulation appears prominent in a narrow frequency band around the perceived vibrotactile frequency. **(B)** *Top*: Average topography of the SSEFs for all f1 stimuli. *Bottom*: Source reconstruction of the mean SSEF, weighted by relative amplitude, thresholded at *p* < 0.001 (uncorrected). Sources span the somatosensory areas 1, 2, and 3b. **(C)** SSEFs expressed as average of channels with strongest response during stimulus presentation (marked with ^∗^ in topography), depicted in the center. The four time-courses show the mean narrow-band power around the frequencies of base (f1) and comparison (f2) stimuli for equal-numbered correct and incorrect trials. The gray shading indicates time points of significant difference between the two subsets from cluster analysis (*p_FWE_* < 0.05). **(D)** Scatterplot of mean narrow-band SSEF amplitude and percent correct responses (PCR) for each subject. The correlation of these two metrics was not significant (*r* = 0.34, *p* = 0.11).

Stimulus evoked MEG activity from all planar gradiometers are depicted in **Figure 2A** for one exemplary stimulus pair (f1 = 23 Hz; f2 = 27 Hz). The vibrotactile stimulus evoked strong frequency-specific steady-state evoked fields (SSEFs), contralateral to the stimulated hand (**Figure 2B**). Source reconstruction localized the steady-state evoked response focally to the right somatosensory cortex, with a cluster spanning areas 3b, 1 and 2 (peak: 24, -38, 57). Crucially, evoked responses were limited to the duration of stimulus presentation and were absent during the retention interval.

We were interested whether subjects’ performance was related to their steady-state evoked responses as previously reported with EEG (Spitzer et al., 2010). **Figure 2C** shows the grand average narrow band evoked activity at the frequency of f1 and f2 stimulation, computed over all stimulus conditions for equal subsets of correct and incorrect trials. The illustrated time-courses are based on averages from planar gradiometers over right somatosensory areas, where SSEFs were most pronounced. Statistical analysis revealed differences between correct and incorrect trials during both base (f1) and comparison (f2) stimulus presentation (*p* < 0.05). This difference is likely due to participants increased attention during correct trials, which has been shown to enhance somatosensory evoked potentials (Bardouille et al., 2010). Additionally, we tested whether individual SSEFs were related to behavioral performance across participants. The correlation between subject’s PCRs and SSEF amplitude was not significant [Pearson’s *r*(21) = 0.34, *p* = 0.11; **Figure 2D**], however, there was a trend toward stronger SSEFs in subjects with higher performance.

### Induced MEG Responses

**FIGURE 3:**
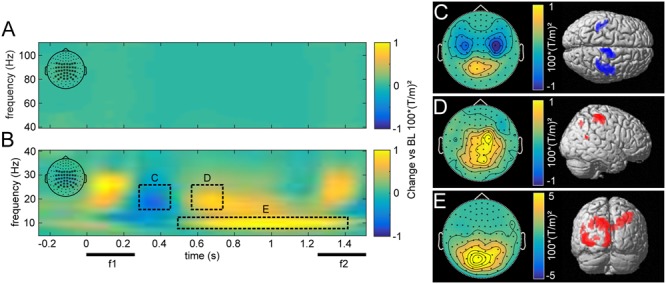
Grand average induced power for high (40–110 Hz; **A**) and low frequencies (5–40 Hz; **B**), compared to pre-stimulus baseline for central parieto-occipital channels as marked in the top left corner. The dashed rectangles illustrate time-frequency windows with increases and decreases induced by stimulus presentation. Beta power first decreased after stimulus presentation **(C)**, then rebounded with an increase in right somatosensory areas, contralateral to the stimulated hand **(D)**. Alpha power was elevated throughout the delay period, most strongly in occipital areas **(E)**. **(C–E)** Show the topographies and source reconstructions (*p* < 0.01, uncorrected) of observed stimulus induced changes.

The overall induced responses observed in higher and lower frequencies pooled over all trials are illustrated in **Figure 3**. Transient and steady-state evoked potentials were eliminated by subtracting the average waveform before time-frequency transformation for each base and comparison frequency pair. Because the piezoelectric stimulation device created an artifact that varied trial-by-trial, subtracting the average waveform left a residual artifact that was restricted to the time of stimulus presentation (**Figure 3B**).

In comparison to a prestimulus baseline, vibrotactile stimulation induced the typically observed changes in the beta band (15–25 Hz) over somatosensory areas (see Spitzer et al., 2010). During and immediately after stimulation, we observed a beta power decrease over bilateral somatosensory channels (**Figures 3B,C**; peak: 42, -26, 52), which was followed by a rebound, dominantly contralateral to the side of stimulation (**Figures 3B,D**; peak: 46, -34, 63). Moreover, alpha band (7–12 Hz) activity was increased during the retention phase in posterior channels (**Figures 3B,E**). Source reconstruction of this effect revealed a distributed activation pattern over visual regions that was most robust ipsilateral to the stimulated hand (peak: -12, -90, 45). Furthermore, this effect was more pronounced in correct than incorrect trials (*p_FWE_* < 0.05). As visual input was inconsequential for task performance during this time, alpha power appears to reflect top-down inhibition of task-irrelevant cortical areas (Klimesch et al., 2007; Jensen and Mazaheri, 2010).

While there were no changes in induced gamma power (>40 Hz, **Figure 3A**) with respect to the prestimulus baseline, frontal gamma power between 70 and 110 Hz was related to task performance. In particular, we found higher broadband gamma band power for correct as compared with incorrect trials (*p_FWE_* < 0.01). However, this effect neither correlated with changes in occipital alpha power across subjects, nor with participants’ overall performance (both *p* > 0.05), as had been reported previously (Haegens et al., 2010).

### Parametric Contrast of Induced Beta Oscillations

**FIGURE 4:**
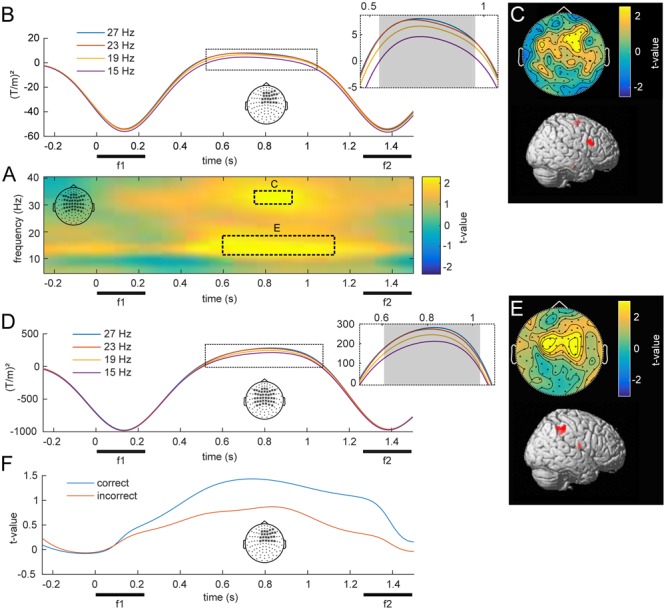
Overview of low frequency oscillations modulated by f1. **(A)** Statistical parametric map of oscillatory power as a function of f1, averaged over bilateral fronto-parietal electrodes denoted in the top left corner. Statistical analysis revealed two separate clusters, one in a higher and one in a lower beta band (*p* < 0.05, FWE), which are marked with dashed lines. **(B)** Illustration of the average power at 30–35 Hz for the four f1 stimuli throughout the retention interval for right frontal channels. The gray area denotes a significant linear trend. **(C)** Topographical scalp distribution and corresponding source reconstruction of the TF cluster in the upper beta band as marked in **(A)**. **(D,E)** Time-course, topography and source reconstruction in analogy to **(B)** and **(C)** for the TF cluster in the lower beta band (10–20 Hz) as depicted in **(A)**. **(F)** Grand average time-course of WM effect in correct and incorrect trials over right frontal channels at 30–35 Hz.

The central aim of this study was to identify changes in oscillatory power that scale with the stimulus held in WM throughout the delay period. **Figure 4** illustrates such a parametric WM effect for low frequencies (5–40 Hz). A cluster-based permutation test revealed TF windows in which the effect was statistically significant (**Figure 4A**). Interestingly, this analysis indicated two distinct clusters in the beta band (both *p_FWE_* < 0.05), centered at the middle of the retention interval. One cluster spanned frequencies in the lower beta band (10–20 Hz) and showed the strongest modulation over bilateral parietal channels (**Figure 4E**). Source localization of this effect indicated focal activity in the right intraparietal sulcus of posterior parietal cortex (PPC; **Figure 4E**; peak: 50, -44, 53), an area closely linked to numerosity processing (Nieder, 2016). Markedly, the average time courses of lower beta power scaled monotonically with the frequency held in WM (**Figure 4D**), as confirmed by linear trend analysis (600–1050 ms, *p* < 0.05). The second cluster extended to the upper beta frequency range (30–35 Hz) and peaked in right frontal channels (**Figure 4C**). The most likely source of this effect was located in the right IFG of the lateral PFC (**Figure 4C**; peak: 48, 12, 35). Similar to the effect in the lower beta band, high beta power scaled with the remembered stimulus frequency throughout a large portion of the retention interval (**Figure 4B**).

To investigate a link to behavior, we compared the observed modulations of beta band power between correct and incorrect trials. When the analysis was based exclusively on incorrect trials, the observed parametric contrast did not reveal any significant effects. However, while analyses of only correct trials revealed the same pattern as the main analysis, the difference between correct and incorrect was not significant. Note that this analysis was limited to a fraction of trials to match the amount of correct with incorrect trials, which strongly reduced statistical power. **Figure 4F** illustrates an example of the performance related differences and displays the parametric contrast statistic at 30–35 Hz for equal-numbered subsets of correct and incorrect trials separately.

### Parametric Modulations of Induced Gamma Activity by f1

**FIGURE 5:**
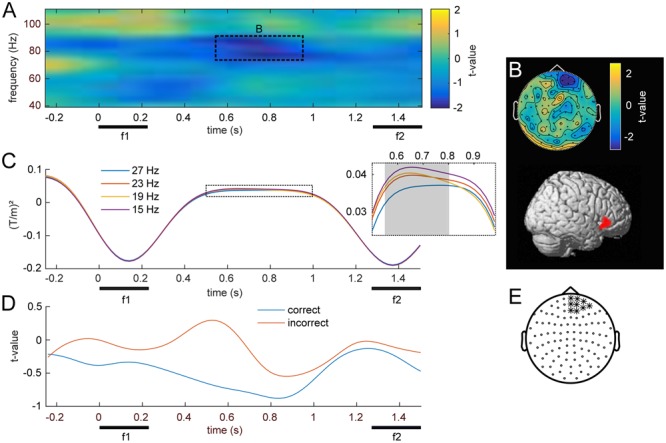
Modulation of induced high frequency activity by f1. **(A)** Statistical parametric map of the linear contrast of the four base (f1) frequencies. The dashed rectangle marks the right frontal cluster (*p* < .05, FWE) for which **(B)** shows the scalp topography and corresponding source reconstruction. **(C)** Time-course of average stimulus induced power for the four different base (f1) stimuli; gray area denotes significant time points. **(D)** Grand-average time courses of gamma band power (74–90 Hz) for equal-numbered subsets of correct and incorrect trials. **(E)** Channels used for **(A–D)**, marked with ^∗^.

The main focus of the present MEG study was the possible parametric modulation of higher frequency oscillations throughout f1 retention, complementing the previously established effects in lower frequencies with EEG. Statistical analysis of frequencies in the gamma band revealed a cluster of prefrontal channels, whose power at 74–90 Hz declined monotonically with increasing f1 frequency (**Figures 5A,E**; *p_FWE_* < 0.05). Source reconstruction of the TF cluster identified the right IFG as the origin of this negative gamma band modulation (**Figure 5B**; peak: 50, -44, 53). In comparison with the high beta effect, which showed the opposite pattern (i.e., an increase with stimulus frequency), the modulation of gamma band activity was localized to more anterior and inferior areas, also reflecting the differences in their respective scalp topographies (viz. **Figures 4C, 5B**). Linear trend analysis of the average power in this frequency range for each of the four f1 stimuli was significant between 550 and 800 ms after f1 onset (**Figure 5C**).

The separate analysis of equal-numbered subsets of correct and incorrect trials resulted in the same pattern as observed in lower frequencies. While an analysis based exclusively on correct trials appeared more similar to the effects of all trials (i.e., showed a modulation by f1), incorrect trials did not show this pattern. However, because this analysis was based upon random permutations of a fraction of trials, statistical power was strongly reduced and no significant differences manifested between the two subsets (**Figure 5D**).

## Discussion

In the present study, we recorded MEG in humans to determine the neural oscillations underlying vibrotactile frequency maintenance during WM. In a sequential frequency comparison task, we identified modulations of spectral power by the to-be-remembered vibrotactile stimulus frequency (i.e., f1) in the beta (at 10–20 and 30–35 Hz) and gamma (at 74–90 Hz) range during the WM period of the task. Oscillatory power in the beta band parametrically increased in parietal and prefrontal areas with the magnitude of f1. In contrast, prefrontal gamma oscillations parametrically decreased with increasing f1.

The sequential frequency comparison task employed in this study required participants to maintain the stimulus frequency of the first stimulus (i.e., f1) in WM. Consistent with previous EEG studies of somatosensory WM (Spitzer et al., 2010; Spitzer and Blankenburg, 2011, 2012) we identified oscillations in the beta band (10–35 Hz) that encoded the frequency of f1 in a parametric manner during the delay period of the task. The parametric change of high beta power was localized to the IFG in full agreement with previous EEG (Spitzer et al., 2010), fMRI (Kostopoulos et al., 2007) and electrophysiological recordings (Romo et al., 1999; Brody et al., 2003) that demonstrated a crucial role of the IFG for parametric somatosensory WM. Contrary to previous EEG recordings (Spitzer et al., 2010; Spitzer and Blankenburg, 2011, 2012), in which the skull typically acts like a low-pass filter (Pfurtscheller and Cooper, 1975), the observed effect extended above 30 Hz and might therefore be termed a change in the gamma, not the beta band.

Interestingly, we also observed modulations of high frequency gamma power in the right IFG. However, this effect displayed the opposite pattern of the parametric modulation of spectral power in the beta band, i.e., gamma band power decreased monotonically with stimulus frequency. The observed effect in the gamma band appeared in the same frequency range (74–90 Hz) as other correlates of WM in MEG (Kaiser et al., 2003; Fuentemilla et al., 2010; Haegens et al., 2010) and was estimated to be located slightly anterior to the high beta band modulation. Whereas the overall induced gamma power was additionally related to performance within subjects, it neither correlated with performance across subjects, nor with alpha power as was previously observed in a similar task by Haegens et al. (2010). The same study also observed a sustained broad band gamma increase in SII during the retention phase for which we found no evidence in the present study. The lack of such a sustained signal favors the notion that WM exhibits dynamic oscillatory changes – not sustained activity – as evidenced in single-cell recordings (cf. Shafi et al., 2007; Stokes et al., 2013; Lundqvist et al., 2016).

As signal detection with MEG depends on large-scale oscillatory changes, we speculate that our observations reflect a population-level correlate of the heterogeneous encoding as a complex pattern of increases and decreases in firing rate observed in single cells (Barak et al., 2010). This is in line with previous EEG studies (Spitzer and Blankenburg, 2011, 2012) hypothesizing that parametric prefrontal WM effects may indicate an abstract internal scaling of analog quantity information, according to task demands. While the basis of this interpretation was confined to prefrontal oscillations in the beta band, the present results extend this view to prefrontal gamma. This is particularly interesting, because gamma amplitudes recorded with EEG, but not beta, have been found to predict neural responses from multiunit activity recordings in monkeys (Whittingstall and Logothetis, 2009), thus being more likely to represent commonalities between monkey and human research.

Contrary to previous EEG studies, we found that low beta band power (10–20 Hz) was also parametrically modulated by the stimulus frequency held in WM. Interestingly, this effect localized to the right intraparietal sulcus (IPS), an area well-established in its role for supramodal number processing (Eger et al., 2003; Castelli et al., 2006; Nieder, 2012). In particular, blood-oxygen-level dependent (BOLD) responses in the IPS can be used for multi-voxel pattern analysis to distinguish between quantities (Eger et al., 2009) and have been shown to activate in conjunction with inferior frontal areas in numerosity tasks (Piazza et al., 2007; Knops et al., 2014). The present results therefore join growing evidence that indicates a common representation of abstract quantity in the IPS and PFC.

It is unclear, however, why previous EEG studies (Spitzer et al., 2010, 2014; Spitzer and Blankenburg, 2011, 2012; Herding et al., 2016) did not detect the observed changes in the IPS. Besides the higher signal-to-noise ratio for shallow sources with MEG compared to EEG, one reason may be that MEG is more sensitive to sulcal than gyral sources, making the detection of oscillations from the intraparietal sulcus more likely than those from, e.g., the IFG (Hämäläinen et al., 1993; Goldenholz et al., 2009).

Notably, the parametric changes in low beta (10–20 Hz) included frequencies as low as those in the alpha range (8–12 Hz – also called ‘mu’), which are commonly associated with the functional disengagement of particular brain areas (Klimesch et al., 2007). However, the low beta signal was parametrically modulated by the stimulus frequency, suggesting a feature-specific role of the underlying neural process. We suggest that our findings may be explained by frequency specific inhibitory processes in sensorimotor areas themselves, as proposed by the discrete coding and periodic replay hypothesis (Sandberg et al., 2003; Lundqvist et al., 2011), and might be an expression of passive maintenance states as theorized by the dynamic coding framework (Stokes, 2015). In agreement with this idea, the observed beta-gamma dynamics may reflect feature specific differences in brief beta and gamma bursts, which would agree with recent observations in monkeys (Lundqvist et al., 2016). Overall, it appears that an intricate interplay of beta and gamma oscillations in fronto-parietal areas underlies tactile WM, as has recently been observed for attention (van Ede et al., 2014).

In summary, we have shown that beta and gamma oscillations in the IFG parametrically encode stimulus features while retaining vibrotactile frequencies in working memory. Interestingly, in contrast to increases in the beta band, gamma oscillations decreased with the to-be-maintained frequency. Additionally, we found a modulation of spectral power by stimulus frequency in a lower frequency range in the intraparietal sulcus, which underlines the close coupling of IPS and IFG for the processing of abstract quantities. Our findings suggest a functional role of neural oscillations for WM in a fronto-parietal network, with an extended role of beta and gamma oscillations for the somatosensory domain.

## Ethics Statement

This study was carried out in conformance with the recommendations of the ethics committee of the Freie Universität Berlin with written informed consent from all subjects and in accordance with the Declaration of Helsinki.

## Author Contributions

AvL, JH, SL, TN, and FB: experiment design, data collection, data analysis, article preparation. BM and AV: data collection, data analysis, article preparation.

## Conflict of Interest Statement

The authors declare that the research was conducted in the absence of any commercial or financial relationships that could be construed as a potential conflict of interest.

## Footnotes

1 http://ohba-analysis.github.io/

2 http://www.fieldtriptoolbox.org

3 www.fil.ion.ucl.ac.uk/spm/
